# Unexposed populations and potential COVID-19 hospitalisations and deaths in European countries as per data up to 21 November 2021

**DOI:** 10.2807/1560-7917.ES.2022.27.1.2101038

**Published:** 2022-01-06

**Authors:** Lloyd A C Chapman, Rosanna C Barnard, Timothy W Russell, Sam Abbott, Kevin van Zandvoort, Nicholas G Davies, Adam J Kucharski

**Affiliations:** 1 Centre for Mathematical Modelling of Infectious Diseases, London School of Hygiene and Tropical Medicine, London, United Kingdom; 2 Department of Infectious Disease Epidemiology, Faculty of Epidemiology and Population Health, London School of Hygiene and Tropical Medicine, London, United Kingdom

**Keywords:** COVID-19, SARS-CoV-2, remaining burden, unexposed population, immunity, vaccination, deconvolution, infection fatality risk, hospitalisations and deaths

## Abstract

We estimate the potential remaining COVID-19 hospitalisation and death burdens in 19 European countries by estimating the proportion of each country’s population that has acquired immunity to severe disease through infection or vaccination. Our results suggest many European countries could still face high burdens of hospitalisations and deaths, particularly those with lower vaccination coverage, less historical transmission and/or older populations. Continued non-pharmaceutical interventions and efforts to achieve high vaccination coverage are required in these countries to limit severe COVID-19 outcomes.

Although many European countries have experienced high burdens of coronavirus disease (COVID-19) and have managed to achieve reasonably high vaccination coverage, there is still a considerable number of people unvaccinated in most countries and it is unclear how many of these individuals have not yet been infected and lack immunity to the virus. Where the number of these unexposed individuals is large, there is the potential for a considerable remaining burden of severe outcomes (hospitalisations and deaths), so it is crucial to quantify how large this remaining susceptible group could be. We therefore estimated the number of unvaccinated and unexposed individuals in 19 European countries based on data available up to 21 November 2021 and introduced a metric to quantify and compare the potential remaining burden of COVID-19 hospitalisations and deaths across countries, accounting for differences in historical burden, vaccine effectiveness and population age structure.

## Determinants of remaining burden

The potential remaining burden of severe COVID-19 disease in a country depends on several factors, but the key determinants are: (i) the proportion of each age group in the population with naturally acquired immunity, (ii) the proportion of each age group with vaccine-induced immunity and (iii) the age structure of the population, which determines the population-level risk of severe outcomes (hospitalisation/death). Estimating the level of naturally acquired immunity in the population from case incidence data is difficult because of variation in reporting levels over the course of the pandemic and across countries. With reliably reported death time series and high-quality estimates of the infection fatality risk (IFR), infection time series can be inferred by deconvolving the death time series with the infection-to-death delay and scaling it by the inverse of the IFR [1,2]. However, since vaccination reduces the risk of death from infection with severe acute respiratory syndrome coronavirus 2 (SARS-CoV-2) and most countries have followed an age-based vaccine roll-out, the IFR for COVID-19 has varied asynchronously between age groups over time, and this needs to be accounted for in the calculation. We therefore used age-stratified death [3-5] and vaccination [6,7] data to infer age-stratified infection time series, accounting for the impact of vaccination on the IFR for each age group over time.

## Vaccination and the infection fatality risk

**Figure 1:**
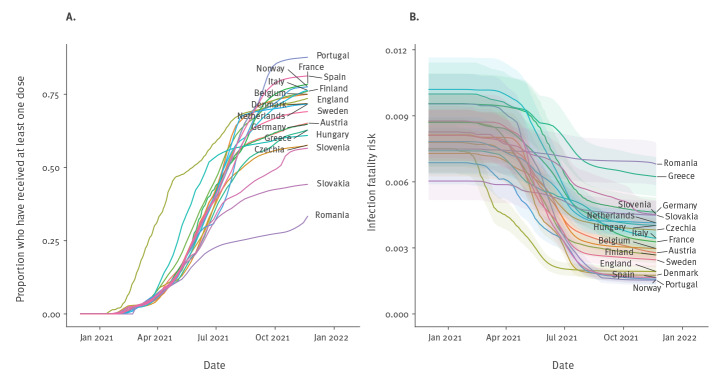
Overall first-dose vaccination coverage (A) and corresponding population-weighted average infection fatality risk (B) over time, 19 European countries, 1 December 2020–21 November 2021

COVID-19 vaccines have been shown to offer strong protection against disease, hospitalisation and death, but weaker protection against infection [8,9]. Their effectiveness against different outcomes also varies according to vaccine type, dose and virus variant [8]. Thus, as vaccination coverage has increased (Figure 1A), the overall IFR has decreased, but in a manner that has depended on the speed and composition of the vaccine roll-out (by age and vaccine product) and the changing proportions of different SARS-CoV-2 variants. We accounted for the varying impacts of vaccination on different disease outcomes by estimating the overall time-varying IFR for each age group for each country as a weighted average of the IFR for unvaccinated individuals and that for vaccinated individuals (accounting for protection against infection from vaccination), averaging the efficacies of different vaccine types and doses against different outcomes for the different circulating variants according to their relative proportions in each country (see Supplement for further details on the time-varying IFR estimation). This resulted in the country-level population-weighted IFRs shown in Figure 1B. The pattern of decrease in the IFR is similar for most countries in Europe, except for England and Romania, where respectively earlier and slower vaccine roll-outs have led to earlier and slower declines. The pre-vaccination IFR was higher (close to 1% vs an average of 0.8%) for countries with older populations such as Italy and Portugal [10].

IFR: infection fatality risk.

Shaded bands in panel B show 95% credible intervals based on a truncated normal approximation to posterior distribution of IFR in [17]. See Supplementary Figure S1 for vaccination coverage and IFR over time by age group for each country.

Source: Vaccination data for European Union countries from the European Centre for Disease Prevention and Control [6] and England from the United Kingdom government COVID-19 dashboard [7].

## Historical burden and naturally acquired immunity

**Figure 2:**
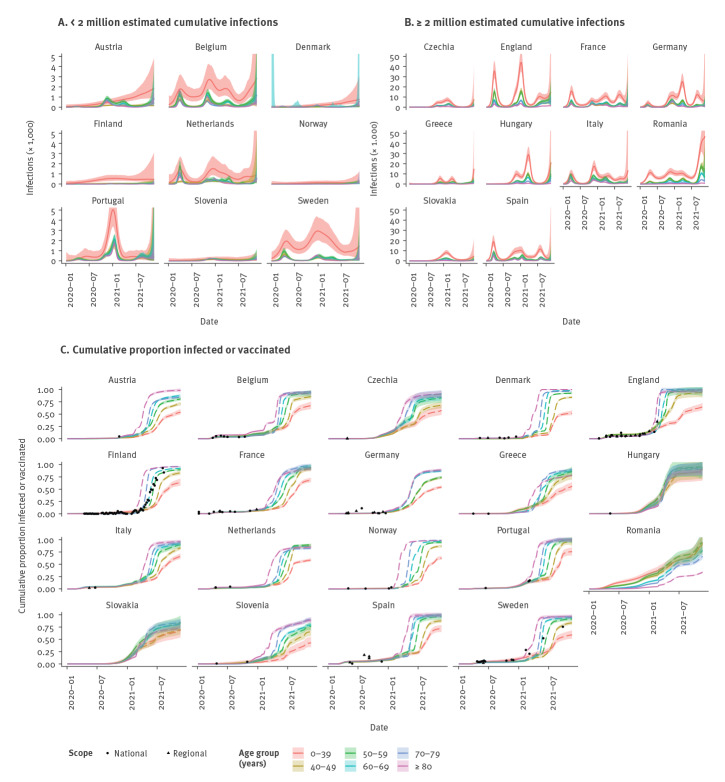
Estimated SARS-CoV-2 infections (A–B) and proportion infected or vaccinated (C) by age over time, 19 European countries, 3 January 2020–21 November 2021

Using the age- and time-dependent IFR for each country (Supplementary Figure S1B), we inferred age-stratified infection time series from age-stratified death time series [11,12] for each country (Figure 2A–B), and from these calculated the cumulative proportion of (unvaccinated and vaccinated) individuals who have been infected and therefore have some degree of immunity. To enable model tractability, we made the simplifying assumption that naturally acquired and vaccine-induced immunity do not wane, given the relatively slow waning of vaccine-induced immunity against hospitalisation and death suggested by current evidence (estimated reductions in protection against hospitalisation and death of 7–8% and 15–18% over 5 months for the Cominarty (BNT162b2 mRNA, BioNTech-Pfizer, Mainz, Germany/New York, United States) and Vaxzevria (ChAdOx1 nCoV-19, Oxford-AstraZeneca, Cambridge, United Kingdom) vaccines respectively [13]) and evidence that reinfections are relatively rare and generally milder than first infections [14]. We assumed that infection with one variant conferred immunity against infection with other variants, given limited observation of immune escape for the SARS-CoV-2 Alpha and Delta variants (Phylogenetic Assignment of Named Global Outbreak Lineages (Pangolin) designation B.1.1.7 and B.1.617.2) [15]. We found considerable variation in the estimated proportion of the population with infection-acquired immunity between countries, ranging from 3% (95% credible interval (CI): 2–5) in Norway over the whole population to 74% (95% CI: 66–80) in Romania.

SARS-CoV-2: severe acute respiratory syndrome coronavirus 2.

Infection time series in panels A–B inferred from age-stratified death time series. Solid lines and shaded bands show maximum a posteriori/median estimates (in panels A–B and C, respectively) and 95% credible intervals for inferred infections; dashed lines in panel B show where vaccination has contributed to immunity. Points in panel C show central estimates from seroprevalence surveys plotted at the mid-point of the survey period, with geographical scope as shown in the legend. In Supplementary Figure S3, the estimated cumulative proportion infected or vaccinated for England is shown against modelled age-stratified seroprevalence estimates from the United Kingdom Office for National Statistics.

Sources: Age-stratified death data from the COVerAGE database [3,4] and INED database [5] (data shown in Supplementary Figure S2). Seroprevalence data from the SeroTracker database [23].

## Vaccine-induced immunity and breakthrough infections

**Figure 3:**
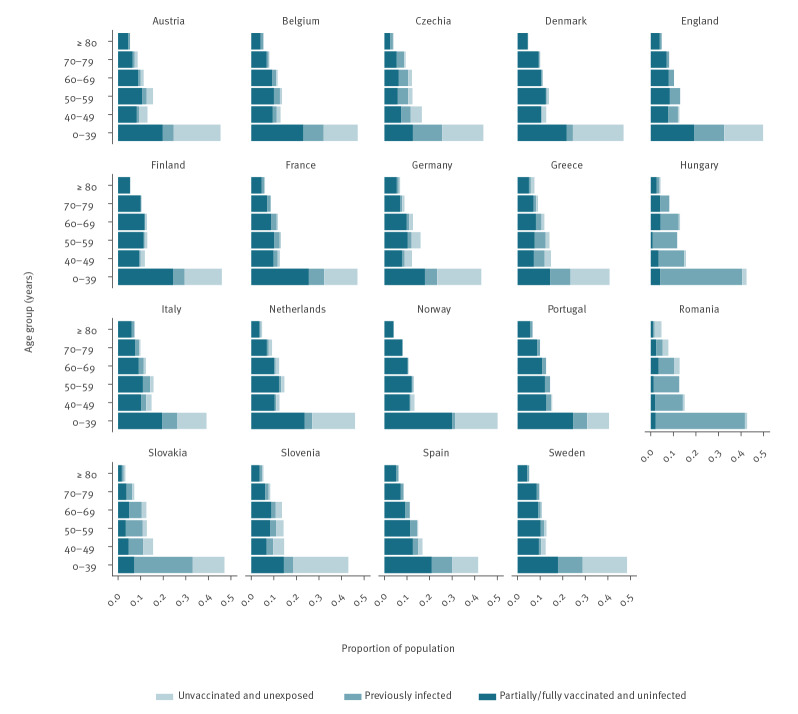
SARS-CoV-2 immune status of the population, 19 European countries, November 2021

To calculate the proportion of the population with immunity acquired through infection or vaccination, or both, it is necessary to account for some infections being breakthrough infections, i.e. among vaccinees, and some vaccinations being given to previously infected individuals, to avoid double-counting these individuals when tallying the overall number with immunity. We did this by solving a difference equation model for the movement of individuals between susceptible, vaccinated and infected states over time in each age group with the back-calculated infection estimates as the input for the number of infections occurring at each time step (see Supplement for equations for the model). The relative proportions of infections at each time step occurring in susceptible and vaccinated individuals were assumed to be proportional to their susceptibility-weighted relative prevalences, and the proportions of vaccinations at each time step given to susceptible and previously infected individuals were assumed to be equal to their relative prevalences among unvaccinated individuals. This approach suggests that in many countries the proportion of individuals in older age groups (≥ 50 years) who were both unvaccinated and unexposed in late November 2021 was small (< 10%) (Figures 2C and 3), and that the majority of unexposed unvaccinated individuals were in younger age groups (< 40 years), with the overall proportion of unvaccinated and unexposed individuals varying from 5% (95% CI: 1–9) in Hungary to 37% (95% CI: 33–39) in Slovenia (see Supplementary Table S4 for estimates for all countries).

SARS-CoV-2: severe acute respiratory syndrome coronavirus 2.

## Remaining hospitalisations and deaths

**Figure 4:**
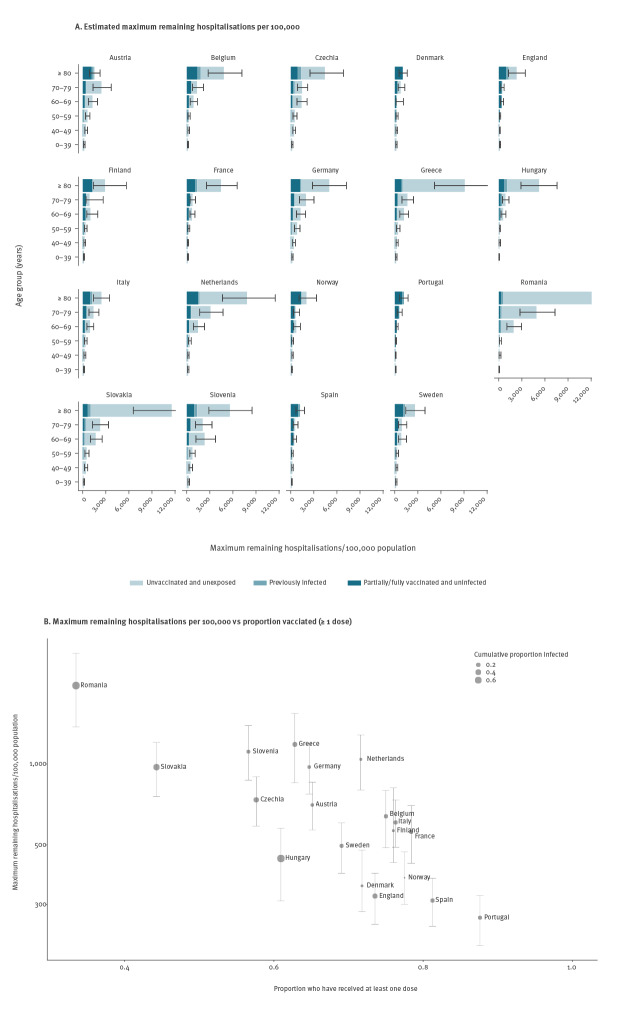
Estimated maximum remaining COVID-19 hospitalisations per 100,000 population by age and immune status (A) and against vaccination coverage (B), 19 European countries, November 2021

Having estimated the remaining number of unvaccinated and unexposed individuals in each age group in each country, we calculated the potential remaining burdens of COVID-19 hospitalisations and deaths as the numbers of hospitalisations and deaths that would occur per 100,000 individuals if the entire population were to be (re-)exposed on 21 November 2021 (Figure 4). In other words, the hospitalisation and death burdens if all unexposed and unvaccinated individuals were infected and a proportion of vaccinated individuals and previously infected individuals were infected dependent on the protection against infection afforded by vaccination and previous infection. These represent upper bounds on the potential burdens of hospitalisations and deaths that could occur in the absence of any further vaccination in the sense that not all unexposed and unvaccinated individuals would become infected in an uncontrolled epidemic [16]. However, they do not account for future waning of immunity, population turnover or potential emergence of immune escape variants, all of which could increase the hospitalisation and death burdens.

COVID-19: coronavirus disease.

Estimation of maximum remaining hospitalisations assumes no waning of immunity or emergence of immune escape variants. Error bars show 95% credible intervals.

Note that the horizontal axis in panel A has been truncated for Greece, Romania and Slovakia to ensure that differences between countries remain visible. An alternative presentation of this plot without truncation of the horizontal axis is provided in Supplementary Figure S6A. The vertical axis in panel B is on a log scale. Supplementary Figure S4 shows the pattern of maximum remaining deaths by age and country and its relationship with vaccination coverage, and Supplementary Figure S5 shows the relationship between remaining hospitalisations and deaths and population age structure.

The estimated maximum overall remaining hospitalisations per 100,000 individuals ranged widely across countries, from 270 (95% CI: 210–320) in Portugal to 2,000 (95% CI: 1,400–2,600) in Romania (see Figure 4B and Supplementary Table S4 for estimates for all countries). This reflects the higher vaccination coverage in Portugal and the lower vaccination coverage, particularly among older individuals, in Romania than in other countries (Figure 3). In absolute terms, the estimated maximum number of remaining hospitalisations ranged from 20,000 (95% CI: 16,000–28,000) for Denmark to 820,000 (95% CI: 650,000–1,000,000) for Germany. 

The comparative pattern of maximum remaining COVID-19 deaths across countries was similar (see Supplementary Figure S4 for potential death burden by age and immune status and its relationship to vaccine coverage), given the strong influence of age on the IFR as well as the infection hospitalisation risk [17,18]. The burden of deaths per 100,000 people ranged from 50 (95% CI: 44–58) for Portugal to 540 (95% CI: 450–630) for Romania, and absolute numbers of deaths ranged from 2,900 (95% CI: 2,600–3,900) for Denmark to 170,000 (95% CI: 150,000–190,000) for Germany. Aside from Romania, countries with a combination of lower vaccination coverage among older age groups, relatively low prior exposure and older populations (Germany, Greece, the Netherlands, Slovenia) had the highest maximum remaining burdens (see Figure 4B and Supplementary Figure S5, which shows the relationship between the potential remaining burden and the proportion of the population aged ≥ 60 years across countries), as they have the potential for much higher numbers of hospitalisations and deaths among the elderly than countries with younger populations and high coverage in older age groups.

## Ethical statement

Ethical approval was not necessary for this modelling study as the analysis uses only publicly available aggregated secondary data.

## Discussion

Our results suggest that the potential remaining burden of COVID-19 hospitalisations and deaths from 21 November 2021 across the 19 European countries considered is substantial, amounting to more than 3 million hospitalisations (95% CI: 2.4–3.8 million) and 640,000 deaths (95% CI: 550,000–750,000), but that it varies considerably between countries, with much higher potential remaining burdens in countries that have experienced less transmission so far, have lower vaccination coverage and/or have older populations. The main driver of the potential remaining burden according to our analysis is vaccination coverage achieved so far, with the proportion of the population infected to date and the age structure of the population having lesser effects, as the speed of the vaccine roll-out has meant that vaccine-induced immunity dominates naturally acquired immunity in most countries. Countries that appear to be at particular risk of high burden include Romania, Greece, Slovenia, the Netherlands, Slovakia and Germany. In contrast, Portugal, Spain, England, Denmark and Norway have a lower estimated risk, under the assumption that vaccines remain effective against circulating variants, because they have much higher vaccination coverage among high-risk groups.

This analysis has a number of limitations (see Supplement for a full discussion). We only consider variation in the IFR that is due to vaccination, but there are several other potential sources of variation, including differences in severity between variants. Rather than an indication of the burden that will actually occur in each country, our estimates provide a measure of the burden that could theoretically still occur in each country if the whole population were simultaneously exposed to SARS-CoV-2 in late November 2021. Our estimates are more relevant to potential short-term burden than long-term burden, as the latter will depend on the impact of waning immunity on severe disease, which is still being determined [13] and will also be influenced by susceptible individuals being replenished through births. For analytical tractability reasons, we did not account for waning of immunity against infection or severe outcomes, the roll-out of boosters or the potential emergence of new variants. Including waning of immunity would increase estimates of potential remaining burden, while boosters would be likely to decrease them, given evidence of increased neutralising antibody titres and protection following booster administration [19-21]. The emergence of new variants, such as Omicron (B.1.1.529), which show immune escape properties [22], could substantially increase future burden and reduce the impact of the proportion vaccinated or previously infected, depending on the extent to which these variants can evade protection against hospitalisation and death provided by vaccination or previous infection.

## Conclusion

Continued non-pharmaceutical interventions and efforts to achieve high vaccination coverage are required in the short term in European countries to limit severe COVID-19 outcomes. Exactly how transmission dynamics evolve in different countries in the longer term will depend on local measures, which could be explored through more detailed country-specific scenario modelling building on this analysis.

## Supplementary Data

2560000 Supplement
